# A novel *Bacillus aerolatus* CX253 attenuates inflammation induced by *Streptococcus pneumoniae* in childhood and pregnant rats by regulating gut microbiome

**DOI:** 10.1007/s00018-024-05232-0

**Published:** 2024-07-29

**Authors:** Ting Yu, Biru Wu, Dimei Zhang, Guanhua Deng, Yi Luo, Ningqianzi Tang, Qiankun Shi, Fang Hu, Guoxia Zhang

**Affiliations:** 1 Department of Environmental Health, Guangdong Provincial Key Laboratory of Tropical Disease Research, School of Public Health, Southern Medical University, Guangzhou, 510515 People’s Republic of China; 2 Guangdong Provincial Key Laboratory of Construction and Detection in Tissue Engineering, Biomaterials Research Center, School of Biomedical Engineering, Southern Medical University, Guangzhou, 510515 China; 3 Key Laboratory of Occupational Environment and Health, Guangzhou Twelfth People’s Hospital, 1Tianqiang St., Huangpu West Ave, Guangzhou, 510620 Guangdong China

**Keywords:** Probiotics, Infection, Gut microbiota, Propionic acid, Butyric acid

## Abstract

*Streptococcus pneumoniae* (*Spn*) is the predominant pathogen responsible for community-acquired pneumonia (CAP) in children under five years old, and it can induce over 17% of pregnant women*.* However, no more effective measures exist to prevent infection induced by *Spn* in these two special populations. The beneficial microbes can antagonize *Spn* and provide new targets for preventing pneumococcal infections. This study used 16S rRNA gene sequencing and targeted metabolomics to evaluate the role of the *Bacillus aerolatus* CX253 (CX253) in alleviating *Spn* infection. Additionally, the colonization of CX253 was observed in nose, trachea, and lung by using confocal laser scanning microscopy and fluorescent labeling techniques. Compared with the model group, the expression level of interleukin-1β was dropped 1.81-fold and 2.22-fold, and interleukin-6 was decreased 2.39-fold and 1.84-fold. The express of tumor necrosis factor-α was down 2.30-fold and 3.84-fold in prevention group of childhood and pregnant rats, respectively. The 16S rRNA sequencing results showed that CX253 administration alone significantly increased the abundance of *Lactobacillus*, *Limosilactobacillus*, and *Prevotella* in the gut of childhood and pregnant rats. Furthermore, the CX253 increased propionate in the gut of childhood rats and increased propionate and butyrate in the gut of pregnant rats to inhibit pulmonary inflammation. In summary, CX253 attenuated *Spn*-induced inflammation by regulating the gut microbiota and SCFAs. The research provides valuable information for the prevention of pneumonia.

**Graphical Abstract:**

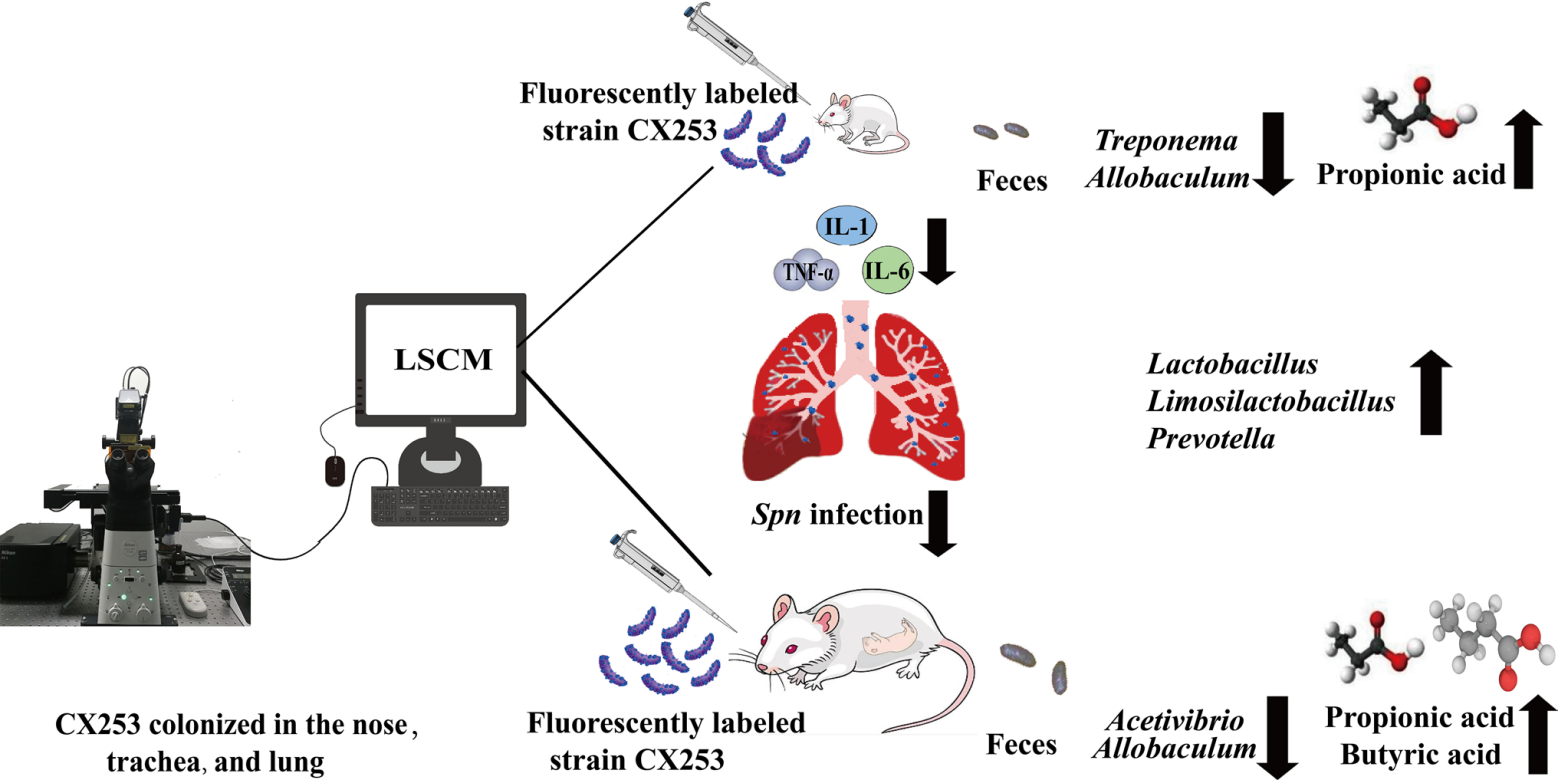

**Supplementary Information:**

The online version contains supplementary material available at 10.1007/s00018-024-05232-0.

## Introduction

According to the World Health Organization (WHO), China has the second highest number of new cases of pneumonia in children, with 21.1 million per year, second only to India [1]. Recent research has revealed that pregnant women with CAP face an increased risk of delivering low-birth-weight babies and premature babies. In more severe cases, respiratory failure and death may occur before or during childbirth [2, 3]. The main pathogen causing pneumonia in pregnancy is *Spn*, and risk factors for its development include anemia and prenatal use of corticosteroids and contraction inhibitors [4]. *Spn* is a Gram-positive bacterium that causes pneumonia with clinical symptoms such as high fever, chills, coughing up sputum, and pleuritic chest pain. It accounts for morbidity and mortality from CAP in children and pregnant women [5, 6]. How does *Spn* affect children and pregnant women? The *Spn* usually colonizes the nasopharyngeal area, especially in childhood, and migrates to normally sterile body parts, resulting in infections such as invasive pneumonia, bacteremia, and meningitis [7]. Additionally, *perinatal Spn* colonization poses a rare yet significant risk factor for severe early-onset sepsis (EOS) among newborns [8]. The risk of *Spn* to these two special populations cannot be ignored, and prevention by pneumococcal conjugate vaccination (PCV) is mostly used at this stage.

Although PCV injection is currently the most common method of preventing *Spn* infection, it has been associated with negative health effects in the specific populations [9]. Studies have demonstrated that PCV-induced antibody immunity is highly serotype-specific and only protects against certain strains included in the vaccine. Furthermore, its effectiveness in preventing pneumonia is lower compared to sepsis and meningitis prevention. In addition, PCV vaccination alters *Spn* serotype ecology and vaccine efficacy decreases over time [10, 11]. Maternal influenza immunization has been found to produce a range of adverse effects on infant growth and induce respiratory disease [12]. Regarding infant protection, there is evidence that transplacental transfer of antibodies may be less efficient when the delivery is closer. The mechanisms behind the female immune response to immunization by gestational age and the optimal timing of maternal immunization are unknown [13, 14]. Polysaccharide antigens inadequately exert long-lasting immune memory responses [15, 16]. Therefore, there is an urgent need for the development of new safe alternative methods specifically tailored for these two populations; numerous studies have shown promising results indicating that probiotics can effectively prevent and treat *Spn* infections.

Probiotics are defined by the WHO as live microorganisms that, when used in appropriate amounts, are beneficial to host health and regulate gut microbial balance [17]. Some probiotics play an important role in protecting the host from infections by enhancing adhesion to the gut mucosa, inhibiting pathogen adhesion, competitively excluding pathogenic microbiota, and synthesizing antimicrobial substances [18]. The rational utilization of probiotics can effectively prevent and treat respiratory infections caused by bacteria and viruses. For instance, nasal administration of *Lactobacillus rhamnosus* CRL 1505 improves T cell-mediated *Spn* infection in malnourished mice [19]. *SFBs* in the gut, when naturally present or introduced by probiotic administration or coexistence with mice, stimulate lung T cell 17 responses and protect against *Spn*-induced infection and death [20]. Intranasal administration of *Lactobacillus casei Shirota* or *Lactobacillus rhamnosus* GG to mice reduced viral titers and attenuated symptoms of influenza virus infection [21]. Furthermore, a combination gavage of CBLEB can modulate inflammatory and metabolic-related pathways in rats to combat *Spn* infection effectively [22]. Additionally, intranasal administration of the probiotic strain *Lactobacillus paracasei* NCC2461 increases lung regulatory T cell numbers while attenuating allergic reactions in a mouse model of allergic airway inflammation [23]. These indicate that probiotics have a promising future as a new measure for preventing lung inflammation and deserve intensive research.

CX253 is an anaerobic, gram-positive, endospore-bearing, rod-shaped strain isolated from a schoolyard bioaerosol and classified in the genus *Rodobacter* [24]. Previous studies have shown that probiotics secrete bacteriocins. Bacteriocin can be used as a colonization peptide to help probiotics colonize specific sites and act as a signal peptide to signal other bacteria or the host immune system [25–27]. The mucosal surfaces of the upper respiratory tract and gut tract are physiologically colonized by their microbiota, and the normal microbiota prevents pneumonia by preventing the colonization of potentially pathogenic bacteria and by modulating the immune response [28, 29]. Healthy microbiota can prevent the carriage of potential pathogens by developing colonization resistance mechanisms [30]. Studies have shown that the presence and abundance of *Corynebacterium*, *Dolosigranulum*, and *Moraxella* are reduced during respiratory tract infections in children. It has been proposed that the colonization of *Corynebacterium* in the upper respiratory tract is inversely proportional to the colonization of *Spn* in this site, thus preventing *Spn* infection [31]. As a potential probiotic candidate, CX253 demonstrated significant inhibition of *Spn* growth in antagonism assays while also exhibiting successful colonization within the respiratory tract thereby attenuating *Spn*-induced lung infections.

The presence of microbial communities in both the lung and gut, which share a common embryonic origin and mucosal immune system, suggests an intrinsic and bidirectional link between the two organs in the context of the gut-lung axis [32]. The gut microbiota acts as a protective mediator and plays a crucial role in host defense against pneumoniae infection [33]. SCFAs are the most common metabolites involved in the maintenance of host immune homeostasis [34]. SCFAs produced by gut microbes through dietary fiber breakdown may modulate immune function and prevent allergic airway inflammation [35]. Moreover, propionate and butyrate have various physiological functions within the gut such as maintaining epithelial barrier integrity and stimulating mucus secretion [36], while also influencing host metabolic activities by acting as a bridge between dietary fiber, commensal microbes, and the host [37]. In addition to their high concentrations in the gut environment, propionate and butyrate can diffuse through the bloodstream to reach distal organs like the lungs where they exert immunomodulatory effects [38]. Acetate has shown to enhance the killing effect of alveolar macrophages against *Spn* [39]. Previous studies have demonstrated that butyrate can mitigate *Spn* infection by regulating Th9 cells [40]. Despite numerous proposals regarding bidirectional communication between the gut and lungs, the exact mechanism remains unclear [41]. Based on above introduction, we hypothesized that CX253 could attenuate *Spn*-induced inflammation by colonizing the nose, trachea, and lungs of childhood and pregnant rats, the gut microbiota and SCFAs were involved in this process.

## Results

### CX253 attenuated Spn infection in childhood and pregnant rats

**Fig. 1:**
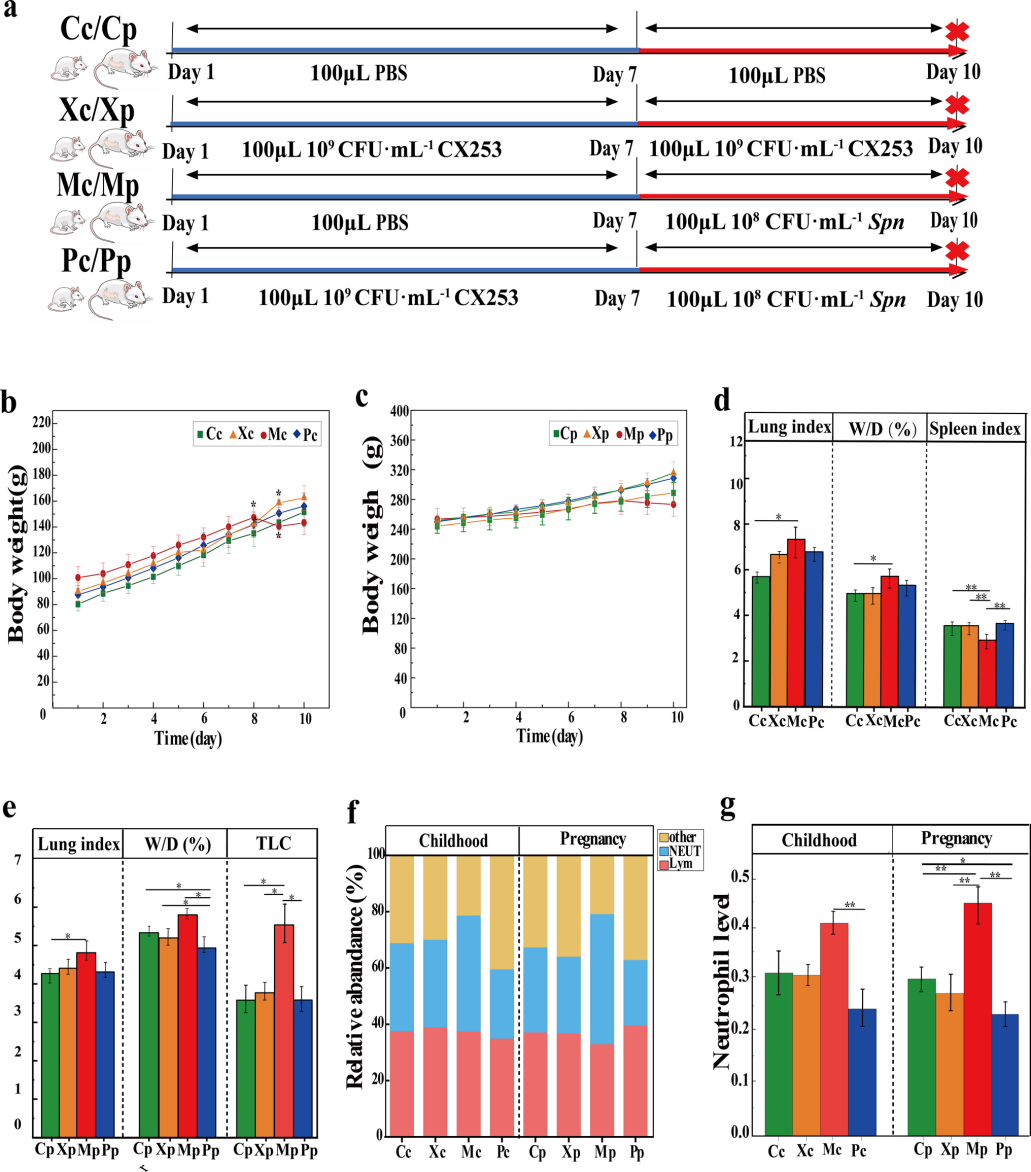
Preventive effect of CX253 against *Spn* infection. **a**: Animal experiments: The childhood rats were divided into control (Cc), CX253 (Xc), model (Mc), and prevention (Pc) groups, Pregnant rats at one week were divided into control (Cp), CX253 (Xp), model (Mp), and prevention (Pp) groups. After one week of acclimatization, Mc/Mp group received one week of nasal drip of PBS, three days of nasal drip of *Spn* on day 8, the Pc/Pp group received 1 week of nasal drip of CX253, and 3 days of nasal drip of *Spn* on day 8, Cc/Cp group nasal drip of PBS, and Xc/Xp group nasal drip of CX253, and all rats were sacrificed on day 10. Trends of body weight of childhood rats (**b**) and pregnant rats (**c**). Lung index, W/D ratio, and Spleen index of childhood rats (**d**). Lung index, W/D ratio, and TLC of pregnant rats (**e**), and leukocyte species in BALF of childhood and pregnant rats (**f**). Neutrophil content in childhood and pregnant rats (**g**). *P*-values: * < 0.05, ** < 0.01 indicate statistical difference

We investigated the role of CX253 during *Spn* infection, 7 days prior to *Spn* (10^8^ CFU mL^–1^) stimulation, SD rats were given CX253 (10^9^ CFU mL^–1^) nasally (Fig. 1a). On the first day of nasal *Spn*, the weight of the Mc group decreased significantly, whereas the weight of the other three groups increased (Fig. 1b). On the first day of nasal *Spn*, the weight of the Mp group decreased while the other three groups continued to increase (Fig. 1c). The lung index of the Mc group was 7.34 ± 0.905, which was higher than the Pc group (6.79 ± 0.503) and the Xc group (6.68 ± 0.348), and was also higher than the Cc group (5.70 ± 0.336). The values of the wet-to-dry weight ratios (W/D) of the Pc and Xc groups were 5.34 ± 0.294 and 4.98 ± 0.394, which were decreased compared with the Cc group, while the W/D value of the Mc group was 5.74 ± 0.472, which was significantly higher than the Cc group. The spleen index of the Mc group was 2.95 ± 0.175, which was significantly lower than the remaining three groups of childhood rats (Fig. 1d). The lung index of the Pp and Xp groups were 4.32 ± 0.157 and 4.41 ± 0.288, respectively, which were not statistically different from the Cp group, while the lung index of the Mp group was 4.81 ± 0.186, which was increased in comparison with the cp group. Compared with the Cp group, the W/D values of the Pp and Xp groups were decreased, whereas the W/D value of the Mp group was 5.80 ± 0.275, which was increased. The total number of leukocytes (TLC) in bronchoalveolar lavage fluid (BALF) was elevated in the Mp group compared to the other three pregnant groups (Fig. 1e). Leukocyte Diff-staining in BALF showed that the proportion of neutrophils in the Mc group was increased compared with the other three groups of childhood rats, and the proportion of neutrophils in the Mp group was increased compared with the other three groups of pregnant rats. However, the proportion was reduced in the Pc group compared with the Cc and Xc groups. The proportion of the Pp group was reduced compared with the Cp and Xp groups (Fig. 1f, g).

### CX253 alleviated lung inflammation in childhood and pregnant rats

**Fig. 2:**
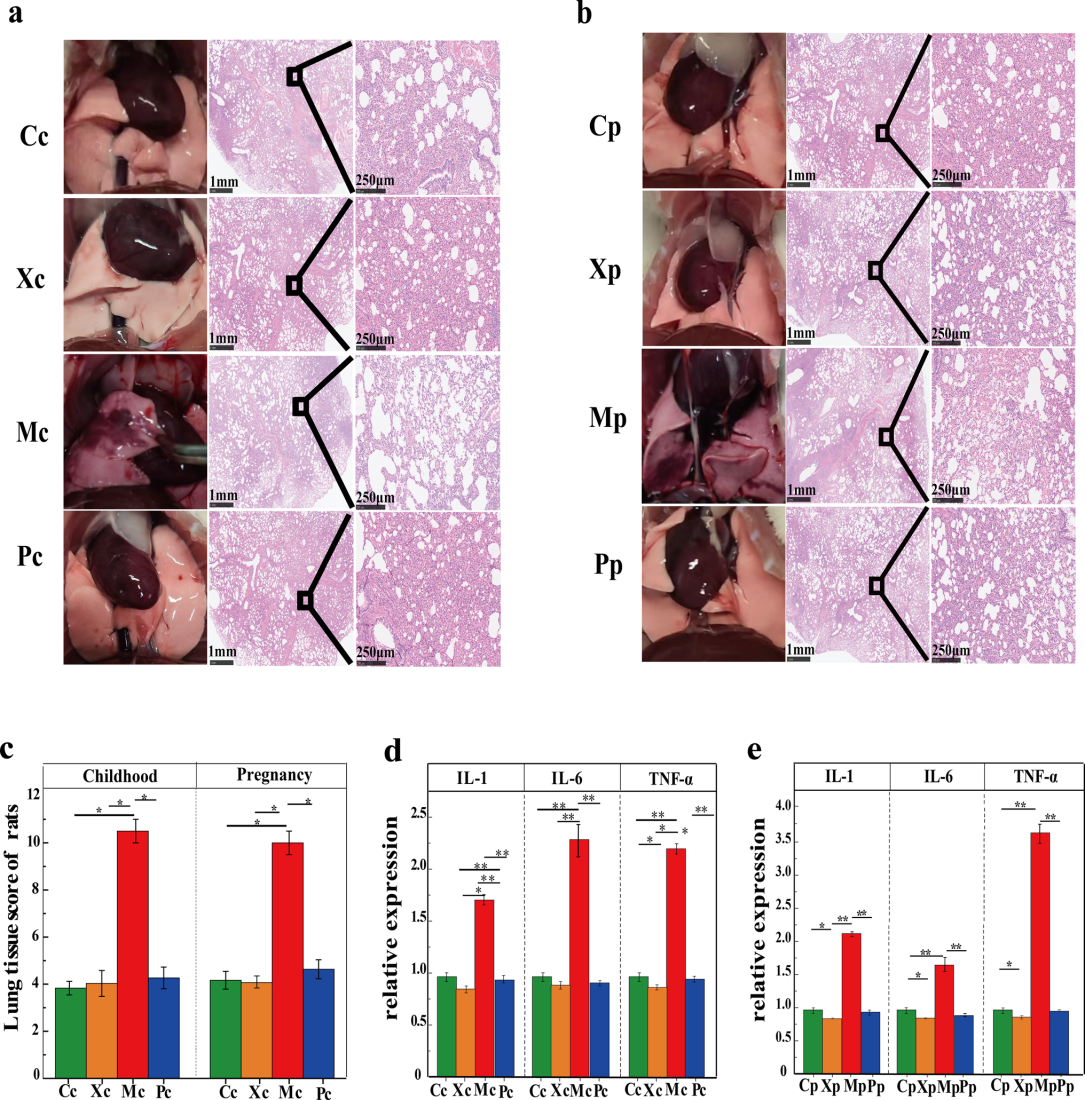
Photographs and original sections of lungs in childhood rats (**a**) and pregnant rats (**b**). The lung-tissue pathological scores of childhood rats and pregnant rats (**c**). Expression of IL-1β, IL-6, and TNF-α in the lung tissues of childhood rats (**d**) and pregnant rats (**e**). *P*-values: * < 0.05, ** < 0.01, indicating statistical difference

The HE-staining sections of childhood rats showed that most of the alveolar wall in the lung tissue of the Mc group was crushed and deformed, lung tissue bleeding, and extensive inflammatory cell infiltration, while the sections of the Pc and Xc groups were similar with the Cc group, did not have these features (Fig. 2a). The HE-staining sections of pregnant rats showed that most of the lung tissue in the Mp group was bleeding, inflammatory cells infiltrated into the alveoli, and the alveolar wall was seriously squeezed and deformed. The lung tissue structure was not damaged in the Pp, Xc, and Cp groups, and no inflammatory cell infiltration was found. (Fig. 2b). According to the lung-tissue pathological score, the Mc group had a higher inflammation score than the Cc, Xc, and Pc groups. The lung-tissue pathological score showed that the Mp group was higher than the Cp, Xp, and Pp groups (Fig. 2c). The IL-1β, IL-6, and TNF-α in lung tissues of childhood rats were decreased in the Pc and Xc groups and increased in the Mc group (Fig. 2d). In addition, the IL-1β, IL-6, and TNF-α in lung tissues of pregnant rats were detected, and these three inflammatory cytokines were reduced in the Xp group, whereas it was increased in the Mp group, and the Pp group was consistent with the Cp group (Fig. 2e).

### Influence of CX253 on gut microbiota alpha diversity and phylum and family level

**Fig. 3:**
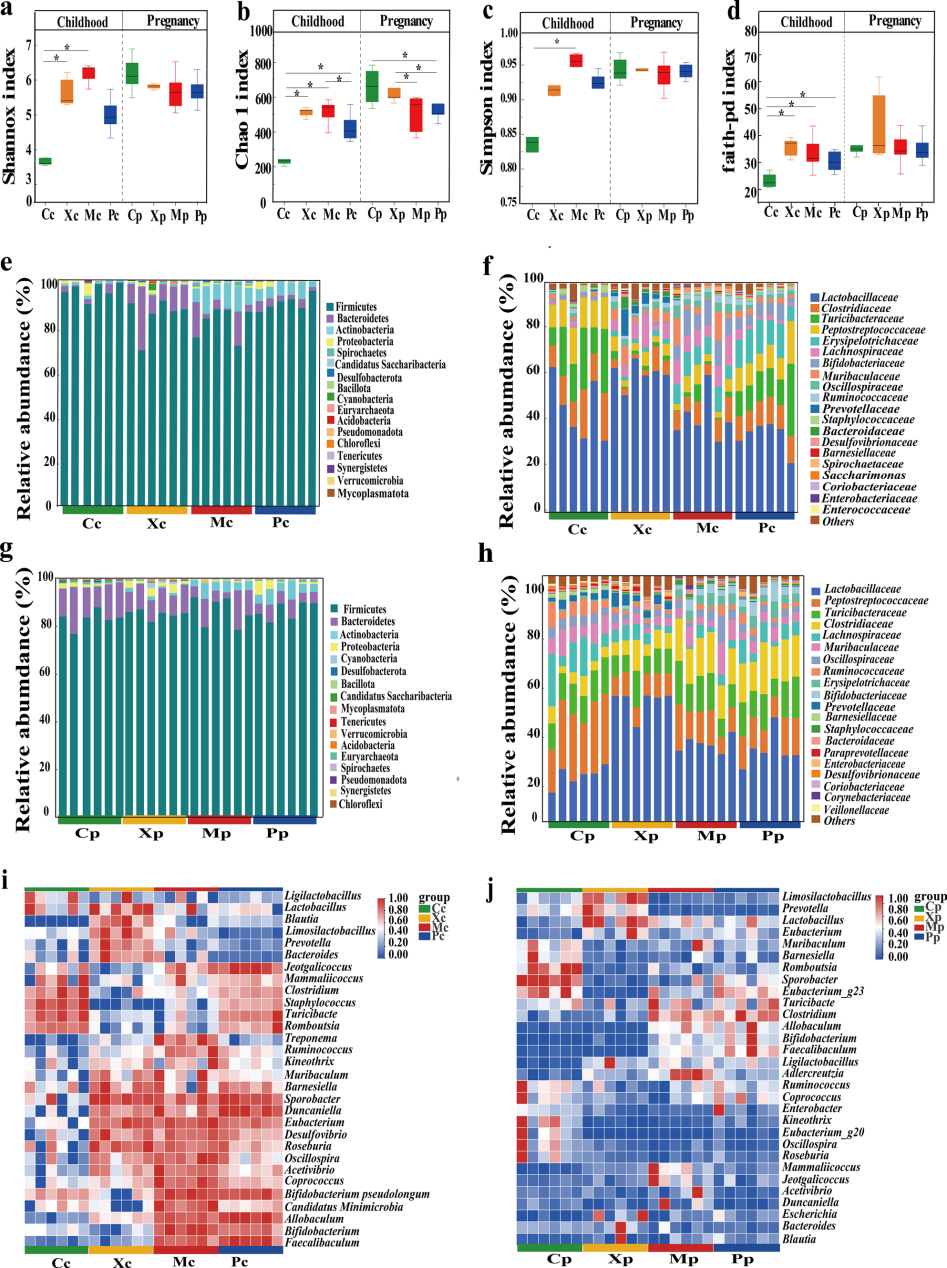
Shannon index of childhood and pregnant rats (**a**), Chao 1 index of childhood and pregnant rats (**b**), Simpson index of childhood and pregnant rats (**c**), and faith-pd index of childhood and pregnant rats (**d**). The composition of phylum in childhood rats (**e**), The composition of family in childhood rats (**f**). The composition of phylum in pregnant rats (**g**). The composition of family in pregnant rats (**h**). The cluster heat map was constructed based on the top 30 bacterial genera in the abundance of childhood rats (**i**) The cluster heat map was constructed based on the top 30 bacterial genera in the abundance of pregnant rats (**j**).* P*-values: * < 0.05, ** < 0.01, indicate statistical difference

The Shannon index of the Mc and Xc groups was higher than the Cc group, while the Shannon index of the four groups of pregnant rats did not differ (Fig. 3a). Compared with the Cc group, the Chao 1 index of the other three groups of childhood rats increased, and the Mc group was the highest. The Chao 1 index of the other three groups of pregnant rats decreased (Fig. 3b). The Simpson index of the Mc group was higher than that of the Cc group, while the Simpson index of the four groups of pregnant rats was not significant difference (Fig. 3c). Compared with the Cc group, the faith-pd index of the other three groups of childhood rats was increased, and there was no statistical difference between the four groups of pregnant rats (Fig. 3d). The abundance of Firmicutes in the Cc group was the highest, while the abundance of Bacteroidetes was lower. Compared with the Cc group, the other three groups of childhood rats had a decrease in Firmicutes and an increase in Bacteroidetes (Fig. 3e). At the family level, childhood rats were predominantly composed of *Lactobacillaceae*, *Clostridiaceae*, *Turicibacteraceae*, *Erysipelotrichaceae,* and *Lachnospiraceae*. The abundance of *Lactobacillaceae* in the Xc group was higher than the other three groups of childhood rats (Fig. 3f). Bacteroidetes were reduced in the Pp group relative to the Cp and Xp groups. There was an increase in Actinobacteria phylum in Mp and Pp groups relative to Cp and Xp groups (Fig. 3g). The top five family levels of pregnant rats were *Lactobacillaceae*, *Peptostreptococcaceae, Turicibacteraceae, Clostridiaceae,* and *Lachnospiraceae*. The abundance of *Lactobacillaceae* in the Xp group was higher than the other three groups of pregnant rats (Fig. 3h).

### Influence of CX253 on gut microbiota genus level and beta diversity

**Fig. 4:**
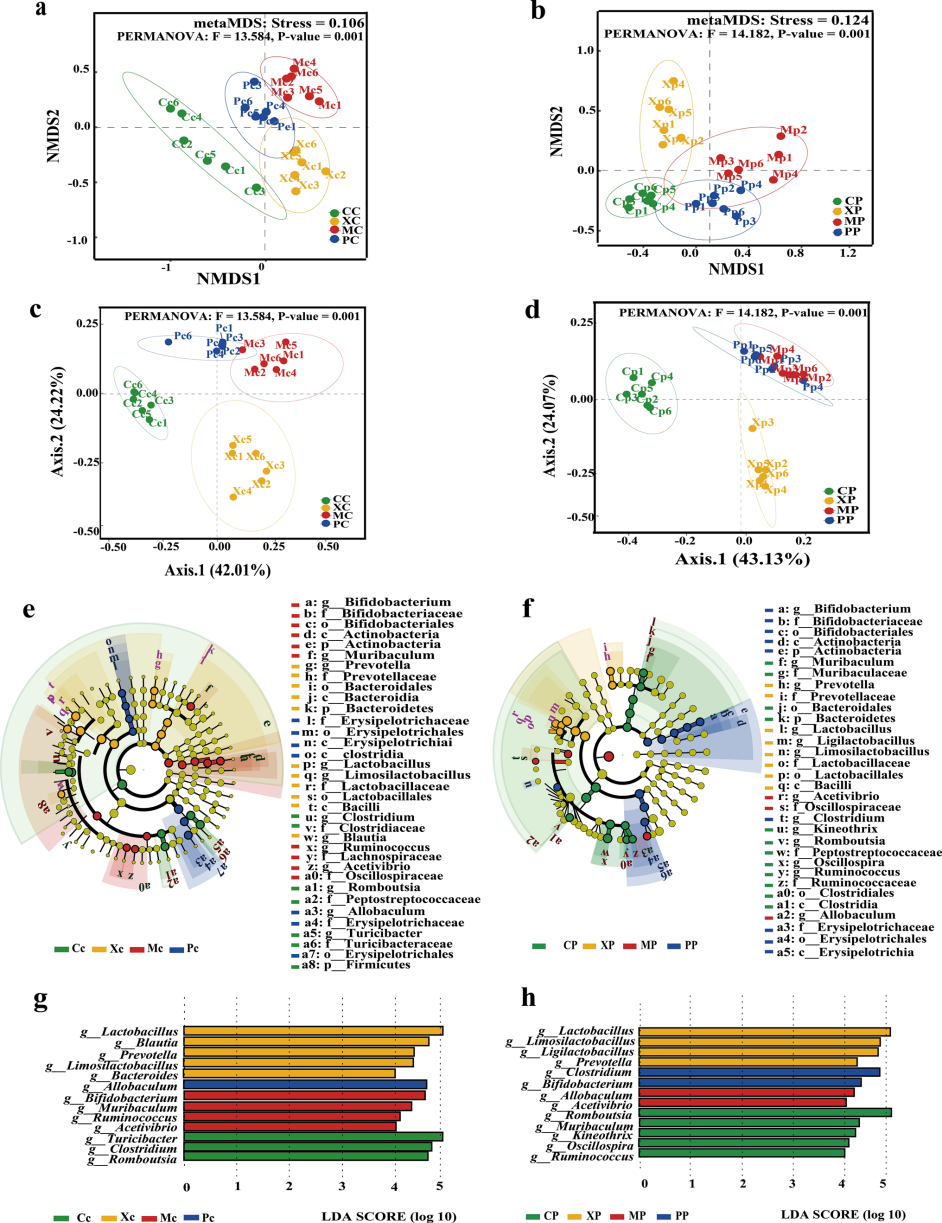
NMDS analysis of childhood rats (**a**) and pregnant rats (**b**). PCoA analysis of childhood rats (**c**) and pregnant rats (**d**). Cladogram for the species differing among groups of childhood rats, with an LDA Score of > 4.5 (**e**), and cladogram for the species differing among groups of pregnant rats, with an LDA Score of > 4 (**f**). Genus’s level LDA Score plot of childhood rats (**g**) and pregnant rats (**h**)

The clustered heat map of the childhood rats revealed the Mc group was positively correlated with *Treponema*, *Acetivibrio*, *Desulfovibrio*, and *Candidatus Minimicrobia*, and the Pc group was negatively correlated with *Blautia*, *Prevotella*, and *Bacteroidetes*. The Xc group was positively correlated with *Limosilactobacillus*, *Prevotella*, and *Lactobacillus* (Fig. 3i). The clustered heat map of pregnant rats showed that the Mp group was positively correlated with *Mammaliicoccus* and *Adlercreutzia*, and the Xp group was positively correlated with *Limosilactobacillus*, *Prevotella*, and *Lactobacillus* (Fig. 3j). NMDS analysis based on the operational taxonomic unit (OTU) bacterial communities showed significant differences between the four groups of childhood and pregnant rats (Fig. 4a, b). The PCoA analysis showed that the percentage of variation explained by PC1 and PC2 explained 24.22% and 42.01% of the variation in childhood rats and 24.07% and 43.13% of the variation in pregnant rats, respectively (Fig. 4c, d). LefSe analyses showed that the gut of the Mc and Mp groups were significantly enriched in *Oscillospiraceae* and *Acetivibrio*, and the gut of the Pc and Pp groups were significantly enriched in *Erysipelotrichaceae* (Fig. 4e, f). In addition, the LDA Score map showed that the enrichment of the Xc group of childhood rats was consistent with that of the Xp group of pregnant rats, which was consistent with the results of the cluster heat map (Fig. 4g, h).

### Influence of gut microbiota on metabolism of SCFAs and the expression of inflammatory cytokines

**Fig. 5:**
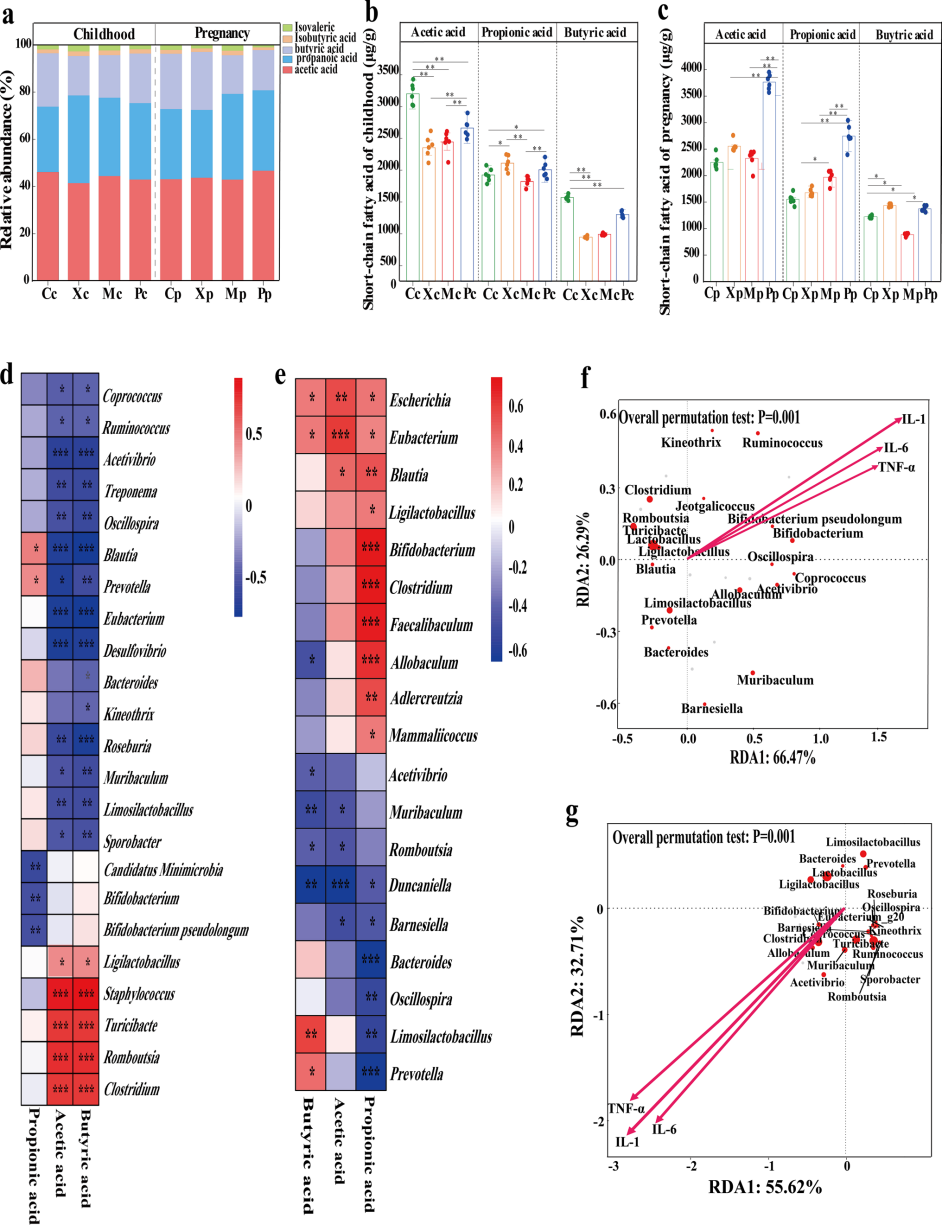
Fecal SCFAs composition of childhood and pregnant rats (**a**). Comparison of SCFA levels in feces of childhood rats (**b**) and pregnant rats (**c**). Correlation heat map between SCFAs and genus of childhood rats (**d**) and pregnant rats (**e**). Redundancy analysis of inflammatory cytokines and genus level of childhood rats (**f**) and pregnant rats (**g**). *P*-values: * < 0.05, ** < 0.01, *** < 0.001 indicate statistical difference

The composition of SCFAs was analyzed in feces of childhood and pregnant rats. The acetic acid was the most abundant, followed by propionic acid and butyric acid (Fig. 5a). The acetic acid and propionic acid and butyric acid in the Mc group were lower than the Cc and Pc groups, and the level of propionic acid in the Xc group was higher than the other three groups of childhood rats (Fig. 5b). The acetic and propionic acid in the Pp group were significantly higher than the other three groups of pregnant rats, and the level of butyric acid in the Pp group was significantly higher than the other three groups of rats (Fig. 5c). The correlation heat map of childhood rats showed that acetic acid and butyric acid were positively correlated with *Clostridium*, *Romboutsia*, *Turicibacte,* and *Staphylococcus*, and propionic acid was positively correlated with *Prevotella* and *Blautia* (Fig. 5d). The correlation heat map of pregnant rats showed that acetic acid was significantly correlated with *Eubacterium* and *Escherichia*, propionic acid was clearly correlated with *Clostridium*, *Faecalibaculum*, and *Allobaculum*, and butyric acid was obviously correlated with *Limosilactobacillius* (Fig. 5e). Meanwhile, the Redundancy analysis showed the IL-1β, IL-6, and TNF-α in the lung tissues of childhood rats were positively correlated with *Bifidobacterium, Bifidobacterium pseudopodium, Allobaculum,* and *Acctivibrio* in the gut. However, these inflammatory cytokines were negatively correlated with *Lactobacillus, Limosilactobacillus, Clostridium, Romboutsia,* and *Prevotella* in the gut (Fig. 5f).*Prevotella*, *Limosilactobacillus*, and *Lactobacillus* were obtuse at the origin line and the arrow, indicating that these bacteria were negatively correlated with the inflammatory cytokines IL-1, IL-6, and TNF-α. Additionally, *Bifidobacterium*, *Turicibacte*, *Allobaculum*, *Clostridium*, and *Acctivibrio* showed acute angles from the origin to the arrow. The results showed that the above bacteria were positively correlated with IL-1, IL-6, and TNF-α (Fig. 5g).

### Real-time quantitative PCR (qPCR) and LSCM were used to detect the colonization of Spn and CX253

**Fig. 6:**
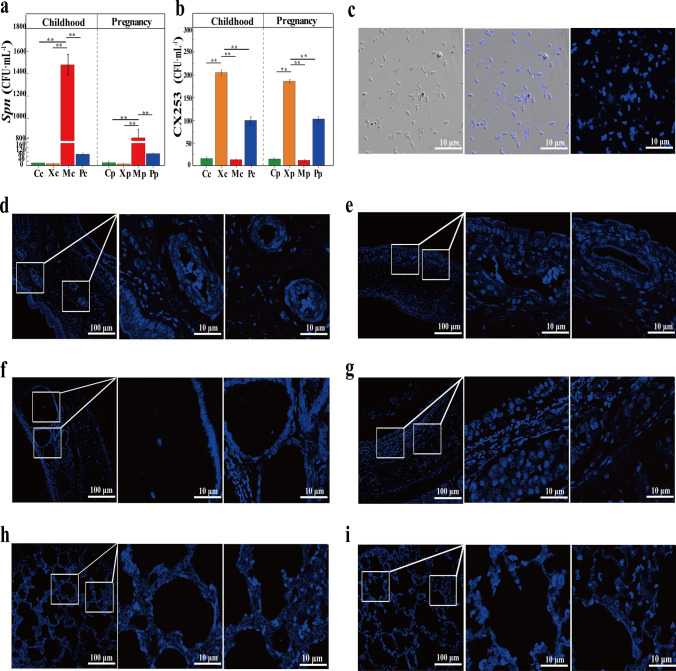
Levels of *Spn* colonization in the lungs of childhood and pregnant rats (**a**) and CX253 colonization in the lungs of childhood and pregnant rats (**b**). labeling of CX253 strain (**c**). LSCM images of nasal sections in childhood rats (**d**) and pregnant rats (e). LSCM images of tracheal sections in childhood rats (**f**) and pregnant rats (**g**). LSCM images of lung tissue sections in childhood rats (**h**) and pregnant rats (**i**)

The qPCR detection of *Spn* colonization in childhood rats showed that the amount of colonization in the lungs of the Mc group was obviously higher than the other three groups, and there was no statistical difference between the other three groups. The results of pregnant rats were consistent with those of childhood rats (Fig. 6a). The qPCR revealed that the number of CX253 colonies in the lungs of Xc and Xp groups were 204.41 ± 6.269 and 185.28 ± 4.534, respectively, which were significantly higher than other three groups. There was no statistical difference between the other three groups. (Fig. 6b). CX253 labeled with chemical materials was observed by LSCM, and plate counting was performed by gradient dilution. The results showed that the material labeling did not affect the growth and division of CX253, and could fluorescently label the colony (Fig. 6c). In addition, LSCM observed the colonization of CX253 in the nasal, tracheal, and lung tissues of both childhood and pregnant rats. In the nasal cavity of childhood and pregnant rats, CX253 was distributed throughout the cilia, plasma glands, and blood vessels of the nasal mucosa (Fig. 6d, e). Part of CX253 was filtered through the nasal cavity and part of CX253 flowed through the trachea with PBS buffer (Fig. 6f, g). Interestingly, we found CX253 eventually deposited in lung tissue, where it was distributed within the alveolar space and around blood vessels (Fig. 6h, i).

## Discussion

In this study, CX253 was clearly colonized in the nose, trachea, and lung. CX253 colonization can significantly reduce the inflammation caused by *Spn* in both childhood and pregnant rats, and gut microbiota plays a major role in this process. CX253 administration alone increased the abundance of *Lactobacillus*, *Limosilactobacillus*, and *Prevotella* in the gut of both childhood and pregnant rats. The early application of CX253 affected the enrichment of *Clostridium* and *Erysipelotrichaceae*. In addition, CX253 intervention exerted their respective anti-inflammatory effects by increasing gut propionate content in childhood rats and increasing gut propionate and butyric acid content in pregnant rats.

CX253 was able to colonize nasal, tracheal and lung tissues of childhood and pregnant rats. Based on LSCM imaging, we found that fractional CX253 was filtered by the nasopharyngeal mucosa, while the rest was eventually deposited in the lung tissue along with the PBS buffer through the trachea. Furthermore, qPCR results demonstrated successful colonization of CX253 in the lungs of childhood and pregnant rats. Notably, CX253 colonization in the respiratory tract significantly downregulated the lung inflammation factors IL-1β, IL-6, and TNF-α in both childhood and pregnant rats, thereby reducing *Spn* infection. An increasing number of studies are now focusing on the colonization of microbes in the nasopharyngeal mucosa and lung tissue, where they interact with long-lived microbes [42]. CX253 is a novel member of the genus *Bacillus* that acts on gut microbes to modulate lung inflammation after colonizing lung tissue, which reflects bidirectional gut-lung communication. It is known that *Bacillus subtilis* exopolysaccharides can effectively reduce airway inflammation in asthmatic mice [43]. Furthermore, oral administration of *Bacillus subtilis* BS50 improves gastrointestinal symptoms in healthy adults [44]. Previous studies have shown that *Bacillus subtilis* not only localizes bacteria to specific ecological niches (such as respiratory mucosa) via bacterial polysaccharides but also directly inhibits gut inflammation and limiting inflammation induced by gut pathogens during infection [45].

We found alterations in the diversity of gut microbiota, including alpha and beta diversity, in childhood and pregnant rats infected with *Spn*. However, intervention with CX253 led to a progression towards a healthy state. Gut microbiome alpha diversity was most significantly elevated in *Spn*-infected childhood rats compared to healthy controls. The Chao 1 index of pregnant rats caused by *Spn* infection was lower than that of the Cp group, and the other indexes were not statistically different. Alpha diversity is strongly correlated with disease state and is the most used indicator for assessing the health of the gut microbiota [46]. However, abnormally elevated alpha diversity does not imply a stable gut microbiota and may suggest a deleterious state with the presence of more opportunistic pathogens [47]. Furthermore, *Spn* infection resulted in significant alterations in gut microbial beta diversity in childhood and pregnant rats, with the greatest differences in gut microbial composition from controls [48], however, the changes in gut microbes caused by CX253 alone or by early administration of CX253 tended to be consistent with healthy controls. Thus, *Spn* infection resulted in profound alterations in the gut microbial structure of both childhood and pregnant rats, possibly due to increased abundance and lower stability of certain opportunistic pathogenic genera in the gut.

The administration of CX253 maintained the homeostasis of gut microorganisms in childhood and pregnant rats, which was strongly associated with an increase in the abundance of probiotics in the gut. At the phylum level, CX253 decreased the Firmicutes/Bacteroidetes (F/B ratio) in childhood rats and increased the F/B ratio in pregnant rats. The F/B ratio provides a critical function for the host, and its size has a profound effect on host metabolism, development, and immune traits [49]. At the family level, early application of CX253 resulted in a higher relative abundance of both *Lactobacillaceae* and *Erysipelotrichiaceae* than the other three groups. *Lactobacillaceae* are well-represented and beneficial bacteria that reduce inflammation [50]. Studies have found a negative correlation between progressive lesion size and *Erysipelotrichiaceae* in patients with Progressive Multiple Sclerosis. *Erysipelotrichiaceae* CCMM is negatively correlated with fatigue, depression, and anxiety, suggesting a potentially beneficial effect [51]. At the genus level, *Treponema* and *Acetivibrio* were significantly enriched in the intestines of childhood and pregnant rats infected with *Spn*. CX253 reduced the enrichment of *Treponema* in childhood rats, *Acetivibrio* in pregnancy rats, and *Allobaculum* in childhood and pregnant rats. Scholars have shown that infections caused by *Treponema* induce the production of inflammatory cytokines and lead to pneumonia [52]. However, administration of CX253 significantly increased the abundance of *Lactobacillus* and *Limosilactobacillus*. *Lactobacillus* and *Limosilactobacillus* are both known to be Gram-positive bacteria and are common components of probiotics [53]. Taxonomic changes in the microbiota (from phylum to genus level) further confirmed the significant regulatory effect of CX253 on gut microbiota.

In the study, CX253 intervention was found to increase propionic acid in the gut of childhood rats, in addition to increasing propionic and butyric acid in the gut of pregnant rats. LefSe analysis showed that CX253 administration resulted in significant enrichment of *Clostridium* and *Erysipelotrichia* in the gut of childhood and pregnant rats. Moreover, the correlation heat map showed that SCFAs were positively correlated with *Clostridium*, *Romboutsia,* and *Turicibacte* in the gut of childhood rats. The gut SCFAs of pregnant rats and *Clostridium*, *Faecalibaculum,* and *Bifidobacterium* was significantly positively related. These suggest that CX253 regulates the abundance of *Clostridium* in the gut of childhood rats and pregnant rats. The *Clostridium* and its sister genus, *Erysipelotrichia*, have a high short-chain fatty acid-producing capacity and can produce butyric acid and propionic acid [54]. Furthermore, they play a key role in the mammalian gut, providing colonization resistance to gut pathogens and promoting immune education in the mammalian host organism. Therefore, we hypothesized that CX253 could also promote propionate and butyric acid production through enrichment of *Clostridium* and *Erysipelotrichia*, thereby reducing lung inflammation, the mechanism of which remains to be verified.

We subsequently observed a significant positive correlation between the inflammatory cytokines IL-1β, IL-6, and TNF-α in the lungs of childhood and pregnant rats with *Allobaculum*, *Acctivibrio*, and *Bifidobacterium* in the gut. Studies have shown that patients with colitis exhibit upregulated IL-1β expression along with an increase in *Allobaculum* and *Bifidobacterium* as well as a decrease in *Lactobacillus* within the gut [55]. Furthermore, a previous study has shown that *Acetivibrio*_*ethanolgignens*_group can be involved in inflammation and lipid metabolism disruption [56]. Conversely, we found a significant negative correlation between these inflammatory cytokines and *Lactobacillus*, *Limosilactobacillus*, and *Prevotella* species; a particularly strong association was observed between IL-1β expression and the gut microbiota. A growing body of evidence supports the important role of *Lactobacillus* in inhibiting inflammatory responses by down-regulating TNF-α [57, 58]. Some scholars have shown that *Prevotella* can produce SCFAs, participate in glucose metabolism, or enhance overall anti-inflammatory effects [59].

## Limitation

We have demonstrated colonization of CX253 in the nose, trachea, and lungs attenuated inflammation induced by *Spn* in childhood rats and pregnant rats. Gut microbes and their metabolites play a major role. However, the mechanism of colonization is unclear. CX253 changes the diversity and composition of gut microbes, increases the abundance of probiotics such as *Lactobacillus* and *Limosilactobacillus*, and induces an increase in propionic acid in the gut of childhood rats and an increase in propionic acid and butyric acid in the gut of pregnant rats. However, the verification in cell, animal level was not conduct. In future studies, we will conduct further research in prevention confirmation and function of CX253 in different population.

## Material and methods

### Bacterial strains and culture conditions

CX253 was isolated from bioaerosols in a school playground in 2019. Strain CX253 was incubated in nutrient solution (NB) (Solarbio, Beijing, China) at 37 ℃, 180 r·min^−1^ for 15 h under shock culture. The absorbance value (OD_600_) of the bacteria detected by spectrophotometer at 600 nm was 1.00, representing about 10^9^ colony-forming units mL^−1^ (CFU mL^−1^). *Streptococcus pneumoniae* GDMCC 1.550 (a type 19 strain) was purchased from the Guangdong Microbial Culture Collection Center (GDMCC). *Streptococcus pneumoniae* was cultured on a blood agar plate (Huankai Microbial, Guangdong, China) at 37 °C, 5% CO_2_, 24 h (Shanghai Buxun Medical & Biological Instrument Co., Ltd). The surface of the blood agar plate was washed with 2 mL of sterile phosphate-buffered alien (PBS, GBico) with pH 7.4. The OD_600_ value of the solution was 0.7, representing about 10^8^ CFU mL^−1^.

### Animals and treatments

3-week-old male Sprague–Dawley rats (n = 32) were Purchased from the Laboratory Animal Management Center of Southern Medical University (Guangzhou, China). The rats were housed under controlled conditions in a specific pathogen-free (SPF) animal laboratory and acclimatized for a week to the start of the experiment. The childhood rats were divided into control (Cc), CX253 (Xc), model (Mc), and prevention (Pc) groups. 7-week-old female SD rats (n = 36) and male SD rats (n = 12) were Purchased from the Laboratory Animal Management Center of Southern Medical University (Guangzhou, China). After a week of adaptation, the female and male rats were mated in a cage at a ratio of 3:1. Pregnant rats (n = 32) at one week were divided into control (Cp), CX253 (Xp), model (Mp), and prevention (Pp) groups. Rats were deeply anesthetized with ether in a confined space to induce infection, and 100 μL bacterial solution containing 1 × 10^8^ CFU mL^−1^ *Spn* was dropped into the left nose to establish an in vivo pneumonia model. Within one week from the beginning of the experiment, 100 μL PBS buffer was dropped in Cc, Mc, Cp, and Mp groups, and 100 μL 1 × 10^9^ CFU mL^−1^ CX253 bacteria was dropped in Pc, Xc, Pp, and Xp groups. On day 8 to day 10 of the experiment, 100 μL PBS buffer was continued to drip in Cc and Cp groups. 100 μL of 1 × 10^8^ CFU mL^−1^ *Spn* was dropped in Pc, Mc, Pp, and Mp groups, respectively, and 100 μL of 1 × 10^9^ CFU mL^−1^ CX253 bacterial solution was continued to drip in Xc and Xp groups. During the three days, rat feces were collected daily and immediately stored at −80 °C. On the 10th day of the experiment, the animals were sacrificed and Serum, lung tissue, cecum, and cecum contents were obtained and stored at −80 ℃.

### Lung index, W/D ratio, and spleen index

The lung index and W/D ratio were used to evaluate the general characteristics of the rats during the experiment. The organ immune index of childhood rats was as follows: spleen index = (spleen weight/body weight of mice) × 10 [60].

### Histopathological observations and lung-tissue pathological scores

Rat lung tissue was fixed and sent to Biossci Biotechnology Co. Ltd, Wuhan, China for preparation of tissue sections and analysis. The lung-tissue pathological scores were determined as previously reported [61].

### Leukocyte species and counts in BALF

After the chest was opened, the left lung was ligated, and the trachea was exposed [62]. With 5 mL syringe, two injections of 5 mL sterile precooling PBS, suction 3 times back and forth, recycling to the sterile centrifuge tube, recovery > 90%. Bronchoalveolar lavage fluid was left for 1 h to visually detect cell precipitation and cell staining, centrifuged at 2000 rpm, 10 min, 4 °C, and then the precipitate was resuspended in 1 mL of precooled sterile PBS. 20 μL cell suspension was placed on the cell counting plate to count TLC. After counting, 100 μL cell suspension was evenly spread on the slide, dried, and fixed in methanol for 20 min, Diff staining was performed according to the instructions of the DIFF-Quik staining kit (Beijing Sorbio Technology Co., LTD.). The percentages of neutrophils, monocytes, lymphocytes, and other white blood cells were counted under microscope after staining.

### RNA extraction and qPCR

0.03 g lung tissue was added to 1 mL of RNAex (Hunan Acres Bioengineering Co., Ltd.) and homogenized with a tissue homogenizer (Shanghai Fluke Technology Development Co., Ltd.). The concentration and purity of RNA extracted by the Trizol method were determined using an ultra-microspectrophotometer K5500, and the integrity of RNA was verified by gel electrophoresis. 500 ng of RNA was reverse transcribed into complementary DNA (cDNA) using Evo M-MLV Reverse Transcription Premix (Guangzhou Ruijin Biotechnology Co., Ltd.) and a PCR instrument (Bio-Rad). The qPCR assays were performed using SYBR Green PCR kit (Guangzhou Ruijin Biotechnology Co., Ltd.) and QuantStudio^™^ 6 Flex qPCR system (Thermo Fisher Scientific), and the QuantStudio^™^ qPCR software was used to compare the cycling threshold (Ct) to calculate the mRNA expression of each group and normalized to glycerol triphosphate dehydrogenase (GAPDH) in the control cDNA samples. The forward and reverse sequences of rat inflammatory cytokines IL-1β, IL-6, and TNF-α are shown in Supplemental Table S1.

### Quantification of Spn infestation

DNA was isolated from *Spn* by the phenol–chloroform method, and the concentration and purity of the DNA obtained were determined by a K5500 ultramicrophotometer (Beijing Corello Technology Development Co., Ltd.). The qPCR was performed on 1, 10^–1^, 10^–2^, 10^–3^, 10^–4^, and 10^–5^ μg of DNA (Forward and reverse sequences of lytA gene are shown in Supplementary Table S2) to detect the expression of lytA in rat lungs, respectively. Scatter plots of DNA concentration (C_DNA_) and qPCR cycle time (Ct) were obtained. The equation of the curve is Y = −3.2276X + 10.5500, R^2^ = 0.9966. Log_10_ C_DNA_ and Ct value were expressed as X and Y, respectively. We summarised the quantitative relationship between *Spn* and Ct value as Y = −3.2276X + 38.4710, R^2^ = 0.9966, and log_10_ CFU and Ct values are denoted as X and Y, respectively [63].

### Quantification of CX253 infestation

DNA was isolated from the CX253 strain by the phenol–chloroform method, and the concentration and purity of the DNA obtained were determined by a K5500 ultramicrophotometer (Beijing Corello Technology Development Co., Ltd.). The qPCR was performed on 1, 10^–1^, 10^–2^, 10^–3^, 10^–4^, and 10^–5^ μg of DNA (Forward and reverse sequences of CX253 are shown in Supplementary Table S2) to detect the expression of CX253 in rat lungs, respectively. Scatter plots of DNA concentration (C_DNA_) and qPCR cycle time (Ct) were obtained. The equation of the curve is Y = −3.8358 X + 3.5500, R^2^ = 0.9966. Log_10_ C_DNA_ and Ct value were expressed as X and Y, respectively. We summarised the quantitative relationship between the CX253 and Ct value as Y = −3.8358X + 25.868, R^2^ = 0.9967, and log_10_ CFU and Ct values are denoted as X and Y, respectively.

### Gut microbial diversity

Total genomic DNA was extracted from stool samples. The V3-V4 hypervariable region of the bacterial 16S rRNA gene was amplified using primers 338F (5′-ACTCCTACGGAGGCAGCAG-3′-) and 806R (5′-GGACTACHVGGTWTCTAAT-3′). The PCR products were extracted from 2% agarose gels, purified using the AxyPrep DNA Gel Extraction Kit (Axygen Biosciences, Union City, CA, USA), and then extracted by QuantiFluor^™^. QuantiFluor^™^ (Promega, USA) for quantification. Purified amplicons were pooled into aliquots for paired-end sequencing on the Illumina MiSeq platform (Illumina, San Diego, USA).

### Extraction and quantification of fecal SCFAs

Fecal samples were randomly selected from six rats in each group for SCFAs extraction. 1 g of feces was collected from each rat and divided equally into two 2 mL centrifuge tubes. 1 mL of precooled PBS was added to each tube, vortexed and shaken for 10 s, and sonicated for 10 min. Then centrifuge at 13,000 rpm, 4 ℃, 10 min, and take the supernatant after centrifugation. The supernatant was collected separately into centrifuge tubes and 10 μL of 50% H_2_SO_4_ solution and 0.5 g of anhydrous calcium chloride were to absorb the water. The supernatant was collected into the injection vial and 10 μL of 250 µg mL^−1^ 2-Ethylbutyric acid was added, and SCFAs were determined and analyzed by GC/MS.

### LSCM imaging and chemical fluorescence labeling

The molar mass of the chemical material is 465.3 g moL^−1^. The synthesis of this material was performed in the following four steps, as shown in Supplementary Fig S1. The mass spectrometry is shown in Supplementary Fig S2a. The UV absorption is shown in Supplementary Fig S2b. The fluorescence spectrum is shown in Supplementary Fig S2c. It was dissolved in 5 mL dimethyl sulfoxide (DMSO) solution according to m = C* M *V (C: molar concentration, M: molar mass, V: solution volume) as the stock solution, and configured with three dose gradients of 1 mmol L^−1^, 2 mmol L^−1^ and 5 mmol L^−1^. 2 μL of the above solution was added to 2 mL of CX253 bacterial solution (OD = 1.0) in the logarithmic growth stage and incubated for 2 h, 4 h, and 6 h. The optimal incubation concentration of the material was determined to be 5 μmol L^−1^, and the optimal incubation time was 4 h. The effect of the chemical material on the growth and division of CX253 was observed under this condition. The diluted 10^3^, 10^4^, 10^5^, and 10^6^ bacterial solutions were coated on the culture dish, respectively, and continued to culture. In addition, the OD_600_ value of bacterial solution after incubation was detected by spectrophotometer, and there was no statistically significant difference.

### Animal labeling experiment

3-week-old male SD rats and 7-week-old male SD rats were purchased from the Laboratory Animal Management Center of Southern Medical University. From the 8th day of adaptation, female and male rats were mated in the same cage at a ratio of 3:1, and the female rats were checked for pregnancy the next morning. 3-week-old childhood rats began labeling experiments on the 8th day of adaptation (n = 8). 2 μL of 5 mmol L^−1^ material was incubated with 2 mL CX253 bacterial solution (OD = 1.0) for 4 h, centrifuged at 4000 rpm, 5 min, 4 ℃, and washed twice with 1 mL sterile PBS solution. The supernatant was discarded, resuspended in the precipitate with 1 mL sterile PBS solution, blown and mixed well, and 100 μL was aspirated with a pipetting pistol and dropped into the left nose of 4-week-old rats for 10 days. On the 11th of the experiment, childhood rats were sacrificed, and nasal, tracheal, and lung tissues were fixed with 4 mL of 4% paraformaldehyde solution. The experiment of pregnant rats was started after one week of pregnancy, and the intervention was consistent with that in childhood rats (n = 8). On the 10th of the experiment, pregnant rats were sacrificed, and nasal, tracheal, and lung tissues were fixed with 4 mL of 4% paraformaldehyde solution. Finally, the samples of childhood and pregnant rats were sent to Guangzhou Kefu Technology Co., LTD., and thick sections were made. Confocal laser scanning microscopy (Nikon, Japan) was used to observe the labeling in vivo.

### Statistical analysis

Data were expressed as mean ± SEM. Kolmogorov–Smirnov and Levene tests were used to test whether the data obeyed a normal distribution and to compare differences in variance between groups. One-way ANOVA analyzed the data that conformed to normality and were variance-aligned. Kruskal-Walli’s test was used for data not following normal distribution. When the variance was uneven between groups, Dunnett T3 was used for two-way comparison between groups. Origin 2018 and Wekemo Bioincloud (https://www.bioincloud.tech/) were used to plot the correlation plots. *P*-values * < 0.05, ** < 0.01, and *** < 0.001 were significantly different.

## Supplementary Information

Below is the link to the electronic supplementary material.

Supplementary file1 (DOCX 22 KB)

## Data Availability

Raw sequence data were uploaded to the National Center for Biotechnology Information Sequence Read Archive SRA database. The data for this study can be accessed at the following link: https://www.ncbi.nlm.nih.gov/sra/?term = PRJNA995926.

## References

[1] Chee E, Huang K, Haggie S, Britton PN (2022) Systematic review of clinical practice guidelines on the management of community acquired pneumonia in children. Paediatr Respir Rev 42:59–68. 10.1016/j.prrv.2022.01.006 35210170 10.1016/j.prrv.2022.01.006

[2] Sheffield JS, Cunningham FG (2009) Community-acquired pneumonia in pregnancy. Obstet Gynecol 114(4):915–922. 10.1097/AOG.0b013e3181b8e76d 19888052 10.1097/AOG.0b013e3181b8e76d

[3] Abu-Raya B, Michalski C, Sadarangani M, Lavoie PM (2020) Maternal immunological adaptation during normal pregnancy. Front Immunol 11:575197. 10.3389/fimmu.2020.575197 33133091 10.3389/fimmu.2020.575197 PMC7579415

[4] Alonso R, Santillan BM, Rodriguez CL, Mainero FA, Oliva V, Venica DP et al (2021) Community acquired pneumonia in patients requiring hospitalization. Medicina 81(1):37–46 33611243

[5] Ashby T, Staiano P, Najjar N, Louis M (2022) Bacterial pneumonia infection in pregnancy. Best Part Res Clin Obstet Gynaecol 85:26–33. 10.1016/j.bpobgyn.2022.07.001 10.1016/j.bpobgyn.2022.07.001 35970746

[6] Kuitunen I, Jaaskelainen J, Korppi M, Renko M (2023) Antibiotic treatment duration for community-acquired pneumonia in outpatient children in high-income countries-a systematic review and meta-analysis. Clin Infect Dis 76(3):e1123–e1128. 10.1093/cid/ciac374 35579504 10.1093/cid/ciac374 PMC9907524

[7] Felix S, Henares D, Munoz-Almagro C, Sa-Leao R (2021) Carriage of multiple *Streptococcus Pneumoniae* capsular types is frequent among children with invasive pneumococcal disease. Eur J Clin Microbiol Infect Dis 40(11):2397–2401. 10.1007/s10096-021-04231-4 33797644 10.1007/s10096-021-04231-4 PMC8017099

[8] Taghivand M, Pell LG, Rahman MZ, Mahmud AA, Ohuma EO, Pullangyeum EM et al (2022) Effect of maternal vitamin D supplementation on nasal pneumococcal acquisition, carriage dynamics and carriage density in infants in Dhaka. Bangladesh BMC Infect Dis 22(1):52. 10.1186/s12879-022-07032-y 35026987 10.1186/s12879-022-07032-y PMC8759256

[9] Rac H, Gould AP, Eiland LS, Griffin B, Mclaughlin M, Stover KR et al (2019) Common bacterial and viral infections: review of management in the pregnant patient. Ann Pharmacother 53(6):639–651. 10.1177/1060028018817935 30556401 10.1177/1060028018817935

[10] Wahl B, O’Brien KL, Greenbaum A, Majumder A, Liu L, Chu Y et al (2018) Burden of *Streptococcus pneumoniae* and *Haemophilus* influenzae type b disease in children in the era of conjugate vaccines: global, regional, and national estimates for 2000–15. Lancet Glob Health 6(7):e744–e757. 10.1016/S2214-109X(18)30247-X 29903376 10.1016/S2214-109X(18)30247-X PMC6005122

[11] Chan W, Entwisle C, Ercoli G, Ramos-Sevillano E, Mcilgorm A, Cecchini P et al (2019) A novel, multiple-antigen pneumococcal vaccine protects against lethal *Streptococcus Pneumoniae* challenge. Infect Immun 87(3):e00846-e918. 10.1128/IAI.00846-18 30530620 10.1128/IAI.00846-18 PMC6386546

[12] Ashby T, Staiano P, Najjar N, Louis M (2010) Bacterial Pneumonia infection in pregnancy. Best Pract Res Clin Obstet Gynaecol 53(2):329–336. 10.1016/j.bpobgyn.2022.07.001 10.1016/j.bpobgyn.2022.07.001 35970746

[13] Gardella B, Dominoni M, Scatigno AL, Cesari S, Fiandrino G, Orcesi S et al (2022) What is known about neuroplacentology in fetal growth restriction and in preterm infants: a narrative review of literature. Front Endocrinol 13:936171. 10.3389/fendo.2022.936171 10.3389/fendo.2022.936171 PMC9437342 36060976

[14] Chen YH, Keller J, Wang IT, Lin CC, Lin HC (2012) Pneumonia and pregnancy outcomes: a nationwide population-based study. Am J Obstet Gynecol 207(4):281–288. 10.1016/j.ajog.2012.08.023 10.1016/j.ajog.2012.08.023 PMC7093888 23021691

[15] Tada R, Suzuki H, Ogasawara M, Yamanaka D, Adachi Y, Kunisawa J et al (2021) Polymeric caffeic acid acts as a nasal vaccine formulation against *Streptococcus Pneumoniae* infections in mice. Pharmaceutics. 10.3390/pharmaceutics13040585 33923897 10.3390/pharmaceutics13040585 PMC8073337

[16] Bondada S, Wu H, Robertson DA, Chelvarajan RL (2000) Accessory cell defect in unresponsiveness of neonates and aged to polysaccharide vaccines. Vaccine 19(4–5):557–565. 10.1016/s0264-410x(00)00161-4 11027821 10.1016/s0264-410x(00)00161-4

[17] Gibson GR, Hutkins R, Sanders ME, Prescott SL, Reimer RA, Salminen SJ et al (2017) Expert consensus document: the international scientific association for probiotics and prebiotics (ISAPP) consensus statement on the definition and scope of prebiotics. Nat Rev Gastroenterol Hepatol 14(8):491–502. 10.1038/nrgastro.2017.75 28611480 10.1038/nrgastro.2017.75

[18] Yuksel N, Gelmez B, Yildiz-Pekoz A (2023) Lung microbiota: its relationship to respiratory system diseases and approaches for lung-targeted probiotic bacteria delivery. Mol Pharm 20(7):3320–3337. 10.1021/acs.molpharmaceut.3c00323 37340968 10.1021/acs.molpharmaceut.3c00323 PMC10324390

[19] Cruz CS, Ricci MF, Vieira AT (2021) Gut microbiota modulation as a potential target for the treatment of lung infections. Front Pharmacol 12:724033. 10.3389/fimmu.2021.635471 34557097 10.3389/fphar.2021.724033 PMC8453009

[20] Felix KM, Jaimez IA, Nguyen TV, Ma H, Raslan WA, Klinger CN et al (2018) Gut microbiota contributes to resistance against pneumococcal pneumonia in immunodeficient rag (-/-) mice. Front Cell Infect Microbiol 8:118. 10.3389/fcimb.2018.00118 29755958 10.3389/fcimb.2018.00118 PMC5932343

[21] Marsland BJ, Trompette A, Gollwitzer ES (2015) The gut-lung axis in respiratory disease. Ann Am Thorac Soc 12:S150–S156. 10.1513/AnnalsATS.201503-133AW 26595731 10.1513/AnnalsATS.201503-133AW

[22] Lv L, Peng L, Shi D, Shao L, Jiang H, Yan R (2022) Probiotic combination CBLEB alleviates *Streptococcus Pneumoniae* infection through immune regulation in immunocompromised rats. J Inflamm Res 15:987–1004. 10.2147/JIR.S348047 35210807 10.2147/JIR.S348047 PMC8857997

[23] Martens K, Pugin B, De Boeck I, Spacova I, Steelant B, Seys SF et al (2018) Probiotics for the airways: potential to improve epithelial and immune homeostasis. Allergy 73(10):1954–1963. 10.1111/all.13495 29869783 10.1111/all.13495

[24] Chen P, Wang D, Ren QQ, Wu J, Jiang Y, Wu Z et al (2020) *Bacillus aerolatus sp*. Nov., A novel member of the genus Bacillus, isolated from bioaerosols in a school playground. Arch Microbiol 202(9):2373–2378. 10.1007/s00203-020-01955-3 32583126 10.1007/s00203-020-01955-3

[25] Zhang Y, Tan P, Zhao Y, Ma X (2022) Enterotoxigenic *Escherichia coli*: intestinal pathogenesis mechanisms and colonization resistance by gut microbiota. Gut Microbes 14(1):2055943. 10.1080/19490976.2022.2055943 35358002 10.1080/19490976.2022.2055943 PMC8973357

[26] Soltani S, Hammami R, Cotter PD, Rebuffat S, Said LB, Gaudreau H et al (2021) Bacteriocins as a new generation of antimicrobials: toxicity aspects and regulations. FEMS Microbiol Rev. 10.1093/femsre/fuaa039 32876664 10.1093/femsre/fuaa039 PMC7794045

[27] Dobson A, Cotter PD, Ross RP, Hill C (2012) Bacteriocin production: a probiotic trait? Appl Environ Microbiol 78(1):1–6. 10.1128/AEM.05576-11 22038602 10.1128/AEM.05576-11 PMC3255625

[28] Man WH, De Steenhuijsen PW, Bogaert D (2017) The microbiota of the respiratory tract: gatekeeper to respiratory health. Nat Rev Microbiol 15(5):259–270. 10.1038/nrmicro.2017.14 28316330 10.1038/nrmicro.2017.14 PMC7097736

[29] Suez J, Zmora N, Segal E, Elinav E (2019) The pros, cons, and many unknowns of probiotics. Nat Med 25(5):716–729. 10.1038/nrmicro.2017.14 31061539 10.1038/s41591-019-0439-x

[30] Thibeault C, Suttorp N, Opitz B (2021) The microbiota in pneumonia: from protection to predisposition. Sci Transl Med. 10.1126/scitranslmed 33441423 10.1126/scitranslmed.aba0501

[31] Horn KJ, Jaberi VA, Arenas V, Andani S, Janoff EN, Clark SE (2021) *Corynebacterium species* inhibit *Streptococcus Pneumoniae* colonization and infection of the mouse airway. Front Microbiol 12:804935. 10.3389/fmicb.2021.804935 35082772 10.3389/fmicb.2021.804935 PMC8784410

[32] Chen J, Zhou D, Miao J, Zhang C, Li X, Feng H et al (2022) Microbiome and metabolome dysbiosis of the gut-lung axis in pulmonary hypertension. Microbiol Res 265:127205. 10.1016/j.micres.2022.127205 36202007 10.1016/j.micres.2022.127205

[33] Han F, Wu G, Zhang Y, Zheng H, Han S, Li X et al (2020) *Streptococcus thermophilus* attenuates inflammation in septic mice mediated by gut microbiota. Front Microbiol 11:598010. 10.3389/fmicb.2020.598010 33384671 10.3389/fmicb.2020.598010 PMC7769777

[34] Martin-Gallausiaux C, Marinelli L, Blottiere HM, Larraufie P, Lapaque N (2021) SCFA: mechanisms and functional importance in the gut. Proc Nutr Soc 80(1):37–49. 10.1017/S0029665120006916 32238208 10.1017/S0029665120006916

[35] Trompette A, Gollwitzer ES, Yadava K, Sichelstiel AK, Sprenger N, Ngom-Bru C et al (2014) Gut microbiota metabolism of dietary fiber influences allergic airway disease and hematopoiesis. Nat Med 20(2):159–166. 10.1038/nm.3444 24390308 10.1038/nm.3444

[36] Liu P, Wang Y, Yang G, Zhang Q, Meng L, Xin Y et al (2021) The role of short-chain fatty acids in intestinal barrier function, inflammation, oxidative stress, and colonic carcinogenesis. Pharmacol Res 165:105420. 10.1016/j.phrs.2021.105420 33434620 10.1016/j.phrs.2021.105420

[37] Morrison DJ, Preston T (2016) Formation of short chain fatty acids by the gut microbiota and their impact on human metabolism. Gut Microbes 7(3):189–200. 10.1080/19490976.2015.1134082 26963409 10.1080/19490976.2015.1134082 PMC4939913

[38] Wang ZJ, Liu J, Li F, Ma SR, Zhao L, Ge P et al (2023) Mechanisms of Qingyi decoction in severe acute pancreatitis-associated acute lung injury via gut microbiota: targeting the short-chain fatty acids-nediated AMPK/NF-κB/NLRP3 pathway. Microbiol spect 11(4):e0366422. 10.1128/spectrum.03664-22 10.1128/spectrum.03664-22 PMC10434154 37338348

[39] Machado MG, Patente TA, Rouille Y, Heumel S, Melo EM, Deruyter L et al (2022) Acetate improves the killing of *Streptococcus Pneumoniae* by alveolar macrophages via NLRP3 inflammasome and Glycolysis-HIF-1α axis. Front Immunol 13:773261. 10.3389/fimmu.2022.773261 35126390 10.3389/fimmu.2022.773261 PMC8810543

[40] Vieira RS, Castoldi A, Basso PJ, Hiyane MI, Camara N, Almeida RR (2019) Butyrate attenuates lung inflammation by negatively modulating TH9 cells. Front Immunol 10:67. 10.3389/fimmu.2019.00067 30761137 10.3389/fimmu.2019.00067 PMC6361737

[41] Principi N, Cozzali R, Farinelli E, Brusaferro A, Esposito S (2018) Gut dysbiosis and irritable bowel syndrome: the potential role of probiotics. J Infect 76(2):111–120. 10.1016/j.jinf.2017.12.013 29291933 10.1016/j.jinf.2017.12.013

[42] Zhang L, Yi H (2022) An exopolysaccharide from *Bacillus subtilis* alleviates airway inflammatory responses via the NF-kB and STAT6 pathways in asthmatic mice. Biosci Rep. 10.1042/BSR20212461 35040955 10.1042/BSR20212461 PMC8799920

[43] Garvey SM, Mah E, Blonquist TM, Kaden VN, Spears JL (2022) The probiotic *Bacillus subtilis* BS50 decreases gastrointestinal symptoms in healthy adults: a randomized, double-blind, placebo-controlled trial. Gut Microbes 14(1):2122668. 10.1080/19490976.2022.2122668 36269141 10.1080/19490976.2022.2122668 PMC9590435

[44] Maguire PT, Loughran ST, Harvey R, Johnson PA (2021) A TLR5 mono-agonist restores inhibited immune responses to *Streptococcus Pneumoniae* during influenza virus infection in human monocytes. PLoS ONE 16(10):e258261. 10.1371/journal.pone.0258261 10.1371/journal.pone.0258261 PMC8513880 34644311

[45] Li Z, Zhou J, Liang H, Ye L, Lan L, Lu F et al (2022) Differences in alpha diversity of gut microbiota in neurological diseases. Front Neurosci 16:879318. 10.3389/fnins.2022.879318 35837118 10.3389/fnins.2022.879318 PMC9274120

[46] Wilmanski T, Rappaport N, Earls JC, Magis AT, Manor O, Lovejoy J et al (2019) Blood metabolome predicts gut microbiome alpha-diversity in humans. Nat Biotechnol 37(10):1217–1228. 10.1038/s41587-019-0233-9 31477923 10.1038/s41587-019-0233-9

[47] Romani L, Del CF, Macari G, Pane S, Ristori MV, Guarrasi V et al (2022) The relationship between pediatric gut microbiota and SARS-CoV-2 infection. Front Cell Infect Microbiol 12:908492. 10.3389/fcimb.2022.908492 35873161 10.3389/fcimb.2022.908492 PMC9304937

[48] Guo Z, Zhang J, Wang Z, Ang KY, Huang S, Hou Q et al (2016) Intestinal microbiota distinguishes gout patients from healthy humans. Sci Rep 6:20602. 10.1038/srep20602 26852926 10.1038/srep20602 PMC4757479

[49] Gonzalez-Lozano E, Garcia-Garcia J, Galvez J, Hidalgo-Garcia L, Rodriguez-Nogales A, Rodriguez-Cabezas ME et al (2022) Novel horizons in postbiotics: *Lactobacillaceae* extracellular vesicles and their applications in health and disease. Nutrients. 10.3390/nu14245296 36558455 10.3390/nu14245296 PMC9782203

[50] Cox LM, Maghzi AH, Liu S, Tankou SK, Dhang FH, Willocq V et al (2021) Gut microbiome in progressive multiple sclerosis. Ann Neurol 89(6):1195–1211. 10.1002/ana.26084 33876477 10.1002/ana.26084 PMC8132291

[51] Watanabe N, Yokoe S, Ogata Y, Sato S, Imai K (2020) Exposure to *Porphyromonas* gingivalis induces production of proinflammatory cytokine via TLR2 from human respiratory epithelial cells. J Clin Med. 10.3390/jcm9113433 33114582 10.3390/jcm9113433 PMC7693763

[52] Duar RM, Lin XB, Zheng J, Martino ME, Grenier T, Perez-Munoz ME et al (2017) Lifestyles in transition: evolution and natural history of the genus *Lactobacillus*. FEMS Microbiol Rev 41:S27–S48. 10.1093/femsre/fux030 28673043 10.1093/femsre/fux030

[53] Wen S, He L, Zhong Z, Zhao R, Weng S, Mi H et al (2021) Stigmasterol restores the balance of Treg/TH17 cells by activating the Butyrate-PPARγ Axis in colitis. Front Immunol 12:741934. 10.3389/fimmu.2021.741934 34691046 10.3389/fimmu.2021.741934 PMC8526899

[54] Sinan C, Haoan Z, Ni C, Wei C (2019) Rape bee pollen alleviates dextran sulfate sodium (DSS)-induced colitis by neutralizing IL-1β and regulating the gut microbiota in mice. Food Res Int 122:241–251. 10.1016/j.foodres.2019.04.022 31229077 10.1016/j.foodres.2019.04.022

[55] Yuan GH, Zhang Z, Gao XS, Zhu J, Guo WH, Wang L et al (2020) Gut microbiota-mediated tributyltin-induced metabolic disorder in rats. RSC Adv 10(71):43619–43628. 10.1039/d0ra07502g 35519721 10.1039/d0ra07502g PMC9058259

[56] Li SH, Hsu WF, Chang JS, Shis CK (2019) Combination of *Lactobacillus* acidophilus and *Bifidobacterium* animalis subsp. lactis shows a stronger anti-inflammatory effect than individual strains in HT-29 Cells. Nutrients. 10.3390/nu11050969 31035617 10.3390/nu11050969 PMC6566532

[57] Jia L, Wu R, Han N, Fu J, Luo Z, Guo L et al (2020) *Porphyromonas gingivalis* and *Lactobacillus rhamnosus* GG regulate the TH17/Treg balance in colitis via tlr4 and tlr2. Clin Transl Immunology 9(11):e1213. 10.1002/cti2.1213 33282294 10.1002/cti2.1213 PMC7685903

[58] Ye Z, Zhang N, Wu C, Zhang X, Wang Q, Huang X et al (2018) A metagenomic study of the gut microbiome in Behcet’s disease. Microbiome 6(1):135. 10.1186/s40168-018-0520-6 30077182 10.1186/s40168-018-0520-6 PMC6091101

[59] Sencio V, Machado MG, Trottein F (2021) The lung-gut axis during viral respiratory infections: the impact of gut dysbiosis on secondary disease outcomes. Mucosal Immunol 14(2):296–304. 10.1038/s41385-020-00361-8 33500564 10.1038/s41385-020-00361-8 PMC7835650

[60] Li Z, Sun Q, Liu Q, Mu X, Wang H, Zhang H et al (2022) Compound 511 ameliorates MRSA-induced lung injury by attenuating morphine-induced immunosuppression in mice via PI3K/AKT/MTOR pathway. Phytomedicine 108:154475. 10.1016/j.phymed.2022.154475 36252465 10.1016/j.phymed.2022.154475

[61] Zhao HY, Chen HG, Meng XY, Yang GT, Hu Y, Xie KL et al (2019) Autophagy activation improves lung injury, and inflammation in sepsis. Inflammation 42(2):426–439. 10.1007/s10753-018-00952-5 30645707 10.1007/s10753-018-00952-5

[62] Fu S, Lu W, Yu W, Hu J (2019) Protective effect of cordyceps sinensis extract on lipopolysaccharide-induced acute lung injury in mice. Biosci Rep. 10.1042/BSR20190789 10.1042/BSR20190789 PMC6591570 31186277

[63] Qin T, Yu T, Liu YQ, Wu JG, Jiang YX, Zhang GX (2023) Roseicella aerolata GB24T from bioaerosol attenuates *Streptococcus Pneumoniae*-introduced inflammation through regulation of gut. Front Microbiol 14:1225548. 10.3389/fmicb.2023.1225548 37547684 10.3389/fmicb.2023.1225548 PMC10397393

