# Correction: A novel *Bacillus aerolatus* CX253 attenuates inflammation induced by *Streptococcus pneumoniae* in childhood and pregnant rats by regulating gut microbiome

**DOI:** 10.1007/s00018-024-05405-x

**Published:** 2024-09-10

**Authors:** Ting Yu, Biru Wu, Dimei Zhang, Guanhua Deng, Yi Luo, Ningqianzi Tang, Qiankun Shi, Fang Hu, Guoxia Zhang

**Affiliations:** 1 Department of Environmental Health, Guangdong Provincial Key Laboratory of Tropical Disease Research, School of Public Health, Southern Medical University, Guangzhou, 510515 People’s Republic of China; 2 Guangdong Provincial Key Laboratory of Construction and Detection in Tissue Engineering, Biomaterials Research Center, School of Biomedical Engineering, Southern Medical University, Guangzhou, 510515 China; 3 Key Laboratory of Occupational Environment and Health, Guangzhou Twelfth People’s Hospital, 1Tianqiang St., Huangpu West Ave, Guangzhou, 510620 Guangdong China

**Correction: Cellular and Molecular Life Sciences (2024) 81:319**

10.1007/s00018-024-05232-0

In this article, Guanhua Deng should have been also denoted as a corresponding author.

In this article the statement in the Funding information section was incorrectly given as

This study was financially supported by the National Science Foundation of China (NSFC 82173476 and 31500076), the Science and Technology Program of Guangzhou, China (201904010161, 104267472039), Guangzhou Municipal Health Commission (Project Number: 20201A010036), and DHEC Research Programme of Occupational Health Pre-control Technology and Management (2024DHEC/PDM163, KJK2024016).

and should have read

This study was financially supported by the National Science Foundation of China (NSFC 82173476 and 31500076), the Science and Technology Program of Guangzhou, China (201904010161, 104267472039), Guangzhou Municipal Health Commission (Project Number: 20201A010036), and DHEC Research Programme of Occupational Health Pre-control Technology and Management (2024DHEC/PDM163, KJK2024016). GD: The method, Resources, validation, survey, Funding access.

The Contributions section was incorrectly given as

TY: Conceptualization, Methodology, Software, Survey, resource, formal analysis, writing, visualization. BW: Methodologies, software, resources, form analysis. DZ: Methodologies, resources, and the investigation. GD, YL, NT, QS: The method, Resources, validation, survey. FH: Resources, review. GZ: editing, oversight, Funding access.

and should have read

TY: Conceptualization, Methodology, Software, Survey, resource, formal analysis, writing, visualization. BW: Methodologies, software, resources, form analysis. DZ: Methodologies, resources, and the investigation. YL, NT, QS: The method, Resources, validation, survey. FH: Resources, review. GD: The method, Resources, validation, survey, Funding access. GZ: editing, oversight, Funding access.

The original article has been corrected.

