# Supplementary material for: A novel Bacillus aerolatus CX253 attenuates inflammation induced by Streptococcus pneumoniae in childhood and pregnant rats by regulating gut microbiome
Source: Cell Mol Life Sci. 2024 Jul 29;81(1):319. doi: 10.1007/s00018-024-05232-0 (PMC11335247; doi:10.1007/s00018-024-05232-0)
Supplement: Supplementary file 1 — Supplementary file1 (DOCX 22 KB) [file 18_2024_5232_MOESM1_ESM.docx]

**A novel *Bacillus aerolatus* CX253 attenuates inflammation induced by *Streptococcus pneumoniae* in childhood and pregnant rats by regulating gut microbiome**

Ting Yu^a^, Biru Wu^b^, Dimei Zhang^a^, Guanhua Deng^c^, Yi Luo^a^, Ningqianzi Tang^a^, Qiankun Shi^b^, Fang Hu^b^*, Guoxia Zhang^a^*

^a^Department of Environmental Health, Guangdong Provincial Key Laboratory of Tropical Disease Research, School of Public Health, Southern Medical University, Guangzhou 510515, PR China.

^b^Guangdong Provincial Key Laboratory of Construction and Detection in Tissue Engineering, Biomaterials Research Center, School of Biomedical Engineering, Southern Medical University, Guangzhou 510515, China.

^c^Key Laboratory of Occupational Environment and Health, Guangzhou Twelfth People's Hospital1Tianqiang St., Huangpu West Ave., Guangzhou, Guangdong, China 510620

*Corresponding authors at:

Department of Environmental Health, Guangdong Provincial Key Laboratory of Tropical Disease Research, School of Public Health, Southern Medical University, Guangzhou 510515, PR China.

Guangdong Provincial Key Laboratory of Construction and Detection in Tissue Engineering, Biomaterials Research Center, School of Biomedical Engineering, Southern Medical University, Guangzhou 510515, China.

E-mail addresses:

[guoxiazhang@smu.edu.cn (G](mailto:guoxiazhang@smu.edu.cn%20(G)uoxia. Zhang)

hufang19@smu.edu.cn (Fang. Hu)

**Supplementary Table**

**Supplementary** **Table 1**. The forward and reverse sequences of rat inflammatory cytokines IL-1β, IL-6, and TNF-α.

| Gene | Forward Sequence | Reverse Sequence |
| --- | --- | --- |
| GAPDH | CTGAACGGGAAGCTCACTGG | TCCGATGCCTGCTTCACTAC |
| IL-1β | GGCTTCCTTGTGCAAGTGTC | AGTCAAGGGCTTGGAAGCAA |
| IL-6 | TCACTGTGCGTTGCAAACAGTGTC | ATACCACAAGGTTGGCAGGTGGAT |
| TNF-α | ATGGGCTCCCTCTCATCAGT | GCTTGGTGGTTTGCTACGAC |

**Supplementary** **Table 2**. Forward and reverse sequences of the lytA gene and CX253

| Gene | Forward Sequence | Reverse Sequence |
| --- | --- | --- |
| lytA | GGTCATGCGAGATACGACAGAA | TCCTCATCAGTCCCAACCAATT |
| CX253 | AGGAACGGAATCAGCCAGCAC | GCCGACGCAAAGTTACCAAA |

**Supplementary Figure**

**Supplementary Fig 1.** The synthesis process of the chemical material. The specific steps are described in the Supplementary Methods.

**Supplementary Fig 2.** The mass spectrometric analysis of the chemical material showed that the relative molecular mass of the material was 465.3 (a). The UV spectrum of the chemical material can specifically analyze the chemical structure of the material (b). The fluorescence spectrum of the chemical material is based on the characteristics and intensity of the fluorescence produced by the photoluminescence phenomenon of the material for qualitative and quantitative analysis (c).

**Supplementary Methods**

**Synthesis Procedures**

Synthesis of 4-bromo-7-(pyridin-4-yl) benzo[c] [1,2,5] thiadiazole (1). 4,7-Dibromobenzo[1,2,5]thiadiazole (4 g, 13.7 mmol), pyridin-4-ylboronic acid (1.68 g, 13.7 mmol), Pd(PPh_3_)_4_ (758.9 mg, 0.68 mmol) were mixed and dissolved in THF (266 mL), stirred under nitrogen atmosphere. Aqueous solution of K_2_CO_3_ (26.6 mL, M) was then injected. The mixture continued to stir and heat at 65 ℃ for 60 h. After cooling to room temperature, the reaction was quenched by water and ethyl acetate, then washed with water (30 mL × 3). The organic phase was dried with Na_2_SO_4_ and the solvent was removed under reduced pressure. The obtained residue was purified with chromatography (hexane/ ethyl acetate = 5/1, v/v) to give compound (1.64 g, 41.2%). ^1^H NMR (400 MHz, CDCl_3_) *δ* 8.77 (s, 2H), 7.98 (d, *J* = 7.5 Hz, 1H), 7.86 (s, 2H), 7.69 (d, *J* = 7.5 Hz, 1H).

Synthesis of 4-(pyridin-4-yl)-7-(thiophen-2-yl) benzo[c] [1,2,5] thiadiazole (2). Compound 1 (1 g, 3.44 mmol), tributyl(thiophen-2-yl)stannane (1.54 g, 4.1 mmol), Pd(PPh_3_)_4_ (190.4 mg, 0.17 mmol) were added into anhydrous toluene, stirred at 80 ℃ for 48 hours under nitrogen atmosphere. After cooling to room temperature, the solvent toluene was concentrated under reduced pressure. The reaction mixture was washed with hexane and dichloromethane several times to extract the liquid phase and dried into solid precipitate. The obtained residue was then purified with chromatography (hexane/ dichloromethane/ ethyl acetate = 3/3/1, v/v/v) to give compound 2 as yellow solid (322.2 mg, 31.8%). ^1^H NMR (400 MHz, CDCl_3_) *δ* 8.77 (s, 2H), 8.17 (s, 1H), 8.00 – 7.78 (m, 4H), 7.50 (s, 1H), 7.24 (s, 1H).

Synthesis of (1-(3-Dimethylaminopropyl)-3-ethylcarbodiimide hydrochloride) (fluorescent material). A dimethylformamide (DMF) solution of Compound 2 (29 mg, 0.1 mmol) and 3-iodopropanoic acid (98.3 mg, 0.49 mmol) were heated at 75 ℃ for 30h. After cooling to room temperature, the reaction mixture was washed with ethyl ether several times and centrifuged for 10 min at 8500 rpm to obtain the precipitate. Then the precipitate was evaporated to dryness on a rotary evaporator and concentrated in vacuo to get compound 3 (52.8 mg). (EDC.HCl) (28 mg, 0.15 mmol) and N-hydroxysuccinimide (NHS) (17 mg, 0.15 mmol) were added into a stirred solution of compound 3 (13 mg, 0.026 mmol) in DMF (2 mL). After stirring the resulting mixture for 3 hours at room temperature, the reaction mixture was washed with ethyl ether several times and centrifuged for 10 min at 8500 rpm to obtain the precipitate. Then the precipitate was evaporated to dryness on a rotary evaporator and concentrated in vacuo (5.8 mg), ESI-MS, m/z: [M-I] ^+^ calcd 465.3, found 465.3.
